# Supplementary material for: BICD Cargo Adaptor 1 (BICD1) Downregulation Correlates with a Decreased Level of PD-L1 and Predicts a Favorable Prognosis in Patients with IDH1-Mutant Lower-Grade Gliomas
Source: Biology (Basel). 2021 Jul 22;10(8):701. doi: 10.3390/biology10080701 (PMC8389329; doi:10.3390/biology10080701)
Supplement: Supplementary file 1 [file biology-10-00701-s001.zip › biology-1274413-supplementary.pdf]

Supplementary Figure S1. The prognostic significance of *HIF1A* expression in the TCGA LGG cohort.

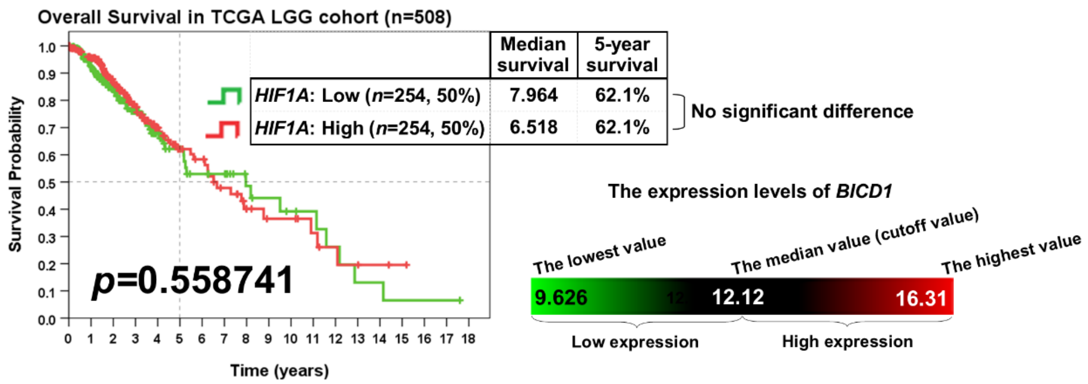

Supplementary Figure S2. The correlation between *BICD1* expression and patient age in LGG patients in the CGGA dataset.

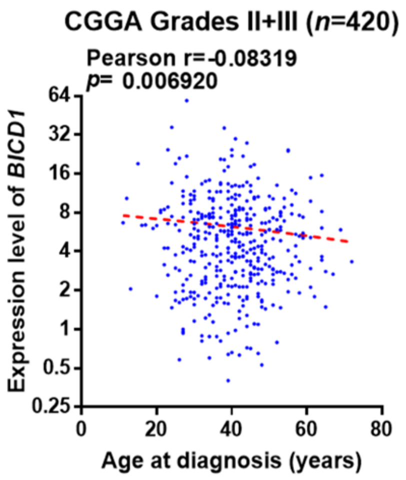

Supplementary Table S1. 34 representative genes associated with the *HIF1A* pathway and their biological effects.

Angiogenesis	Growth and Survival	Glucose metabolism	Invasion and Metastasis	HIF1A activity	Miscellaneous
VEGFA	*EPO*	*ENO1*	*KRT19*	*VHL*	*CA9*
ENG	*ADM*	*SLC2A1 (GLUT1)*	*VIM*	*SUMO1*	*DDIT4*
LRP1	*IGFBP1*	*GAPDH*	*COL5A1*	*MTOR*	*PDCD1(PD-1)*
	IGFBP2	*LDHA*	*FN1*	*HGF*	*CD274(PD-L1)*
	IGFBP3	*HK1*		*MET*	
	TGFA	*HK2*		*BICD1*	
	TGFB3	*ALDOA*			
	TGFB1				
	NOS2				
	IGF2				
	PGK1				
	PDGFB				
	CXCR4				

Supplementary Table S2. Correlations of *BICD1* expression with the clinicopathological features of patients in the TCGA LGG cohort

Clinicopathological feature		***n* (%)**	***BICD1* expression, *n* (%)**		***p***
		**508 (100)**	**Low, *n*=254 (50.0)**	**High, *n*=254 (50.0)**	
***IDH1* status**					2.235×10^{-12}
	Mutant	394 (77.6)	230 (58.4)	164 (41.6)	
	Wild-type	114 (22.4)	24 (21.1)	90 (78.9)	
**Overall survival indicator**					2.649×10^{-10}
	0 (alive)	385 (75.8)	223 (57.9)	162 (42.1)	
	1 (dead)	123 (24.2)	31 (25.2)	92 (74.8)	
***ATRX* status**					4.015×10^{-8}
	Mutant	192 (37.8)	126 (65.6)	66 (34.4)	
	Wild-type	316 (62.2)	128 (40.5)	188 (59.5)	
**WHO Grade**					6.085×10^{-8}
	Grade II	245 (48.2)	153 (62.4)	92 (37.6)	
	Grade III	263 (51.8)	101 (38.4)	162 (61.6)	
***TP53* status**					9.997×10^{-8}
	Mutant	246 (48.4)	153 (62.2)	93 (37.8)	
	Wild-type	262 (51.6)	101 (38.5)	161 (61.5)	
***EGFR* status**					0.000012
	Wild-type	473 (93.1)	249 (52.6)	224 (47.4)	
	Mutant	35 (6.9)	5 (14.3)	30 (85.7)	
**Age at initial diagnosis**					0.003402
	≤40	249 (49.0)	141 (56.6)	108 (43.4)	
	>40	259 (51.0)	113 (43.6)	146 (56.4)	
**1p19q copy number**					0.398106
	Codeleted	171 (33.7)	81 (47.4)	90 (52.6)	
	Others	337 (66.3)	173 (51.3)	164 (48.7)	
**Histological type**					0.854793
	Others	316 (62.2)	159 (50.3)	157 (49.7)	
	Astrocytoma	192 (37.8)	95 (49.5)	97 (50.5)	
**Gender**					0.858284
	Female	226 (44.5)	112 (49.6)	114 (50.4)	
	Male	282 (55.5)	142 (50.4)	140 (49.6)	

Supplementary Table S3. Correlations of the KPS with *BICD1* expression and the clinicopathological features of patients in the TCGA LGG cohort

**Clinicopathological features**	***n***	**KPS (range: 40~100), *n* (%)**		***p***
	**300**	**>80, 197 (65.7)**	**≤80, 103 (34.3)**	
***IDH1* status**				**0.000148**
Mutant	233	166 (71.2)	67 (28.8)	
Wild-type	67	31 (46.3)	36 (53.7)	
***EGFR* status**				**0.002639**
Wild-type	278	189 (68.0)	89 (32.0)	
Mutant	22	8 (36.4)	14 (63.6)	
***BICD1* expression**				**0.005164**
Low	150	110 (73.3)	40 (26.7)	
High	150	87 (58.0)	63 (42.0)	
**Overall survival indicator**				**0.018944**
0 (alive)	212	148 (69.8)	64 (30.2)	
1 (dead)	88	49 (55.7)	39 (44.3)	
**Age (at initial diagnosis)**				0.071784
≤40	138	98 (71.0)	40 (29.0)	
>40	162	99 (61.1)	63 (38.9)	
***TP53* status**				0.096064
Mutant	151	106 (70.2)	45 (29.8)	
Wild-type	149	91 (61.1)	58 (38.9)	
**WHO grade**				0.102078
Grade II	136	96 (70.6)	40 (29.4)	
Grade III	164	101 (61.6)	63 (38.4)	
**1p19q copy number**				0.344430
Codeleted	98	68 (69.4)	30 (30.6)	
Others	202	129 (63.9)	73 (36.1)	
***ATRX* status**				0.477685
Mutant	119	81 (68.1)	38 (31.9)	
Wild-type	181	116 (64.1)	65 (35.9)	
**Gender**				0.963789
Female	148	97 (65.5)	51 (34.5)	
Male	152	100 (65.8)	52 (34.2)	

KPS: Karnofsky Performance Score

Supplementary Table S4. Univariate Cox's regression analysis for comparing the prognostic significance between *BICD1* expression and the clinicopathological features of patients in the TCGA LGG cohort.

Variables	Subgroups	Univariate		
		HR	95% CI	*p*
***EGFR* status**	Mutant (*n*=35) vs. Wild-type (*n*=473)	**5.162**	3.238-8.228	**5.215×10^{-12}**
***IDH1* status**	Wild-type (*n*=114) vs. Mutant (*n*=394)	**4.445**	3.085-6.405	**1.205×10^{-15}**
**WHO grade**	G3 (*n*=263) vs. G2 (*n*=245)	**3.314**	2.231-4.922	**2.960×10^{-9}**
**Age**	>40 (*n*=259) vs. ≤40 (*n*=249)	**2.889**	1.964-4.249	**7.057×10^{-8}**
***BICD1* expression**	High (*n*=254) vs. Low (*n*=254)	**2.731**	1.814-4.113	**0.000002**
**1p19q copy number**	Others (*n*=337) vs. Codeleted (*n*=171)	**2.602**	1.626-4.165	**0.000067**
***TP53* status**	Wild-type (*n*=262) vs. Mutant (*n*=246)	1.422	0.996-2.030	0.052633
***ATRX* status**	Wild-type (*n*=316) vs. Mutant (*n*=192)	1.408	0.975-2.034	0.067904
**Gender**	Male (*n*=282) vs. Female (*n*=226)	1.060	0.741-1.516	0.750157

HR: hazard ratio, CI: confidence interval

Supplementary Table S5. Multivariate Cox's regression analysis for comparing the prognostic significance between *BICD1* expression and the clinicopathological features of patients in the TCGA LGG cohort.

**Variables**	**Subgroups**	**Multivariate**		
		**Adjusted HR**	**95% CI**	***p***
**1p19q copy number**	Others (*n*=337) vs. Codeleted (*n*=171)	**3.787**	2.005-7.154	**0.000041**
**Age**	>40 (*n*=259) vs. ≤40 (*n*=249)	**2.673**	1.726-4.140	**0.000011**
**WHO grade**	G3 (*n*=263) vs. G2 (*n*=245)	**2.201**	1.430-3.387	**0.000335**
***BICD1* expression**	High (*n*=254) vs. Low (*n*=254)	**1.896**	1.219-2.950	**0.004547**
***IDH1* status**	Wild-type (*n*=114) vs. Mutant (*n*=394)	1.687	0.929-3.065	0.086014
***TP53* status**	Wild-type (*n*=262) vs. Mutant (*n*=246)	1.601	0.824-3.110	0.164745
**Gender**	Male (*n*=282) vs. Female (*n*=226)	1.384	0.953-2.010	0.088048
***ATRX* status**	Wild-type (*n*=316) vs. Mutant (*n*=192)	1.141	0.620-2.100	0.672265
***EGFR* status**	Mutant (*n*=35) vs. Wild-type (*n*=473)	1.054	0.591-1.882	0.857723

HR: hazard ratio, CI: confidence interval
